# Editorial: Improving voice outcomes after thyroid surgery and ultrasound-guided ablation procedures

**DOI:** 10.3389/fsurg.2022.1064768

**Published:** 2022-12-12

**Authors:** Che-Wei Wu, Gianlorenzo Dionigi, Kyung Tae

**Affiliations:** ^1^ Department of Otorhinolaryngology-Head and Neck Surgery, Kaohsiung Medical University Hospital, College of Medicine, Faculty of Medicine and Post-Baccalaureate Medicine, Kaohsiung Medical University, Kaohsiung, Taiwan; ^2^ Department of Pathophysiology and Transplantation, University of Milan, Milan, Italy; ^3^ Division of Surgery, Istituto Auxologico Italiano, Istituto di Ricovero e Cura a Carattere Scientifico (IRCCS), Milan, Italy; ^4^ Department of Otorhinolaryngology-Head and Neck Surgery, College of Medicine, Hanyang University, Seoul, South Korea

**Keywords:** thyroid surgery, ultrasound-guided ablation, dysphonia, recurrent laryngeal nerve, vocal fold paralysis, voice, intraoperative neuromonitoring, external branch of superior laryngeal nerve

**Editorial on the Research Topic** Improving voice outcomes after thyroid surgery and ultrasound-guided ablation procedures

Thyroid nodules are a common clinical problem, and thyroid surgery remains one of the more common head and neck procedures (1). In recent years, the field of thyroid procedures for benign and malignant thyroid nodules has expanded from the traditional open-neck approach to include remote robotic or endoscopic access techniques (2) as well as minimally invasive ultrasound (US)-guided ablation procedures, including ethanol ablation (EA), radiofrequency ablation (RFA), microwave ablation (MWA), laser ablation (LA), and high-intensity focused ultrasound (HIFU) (3).

**Figure 1:**
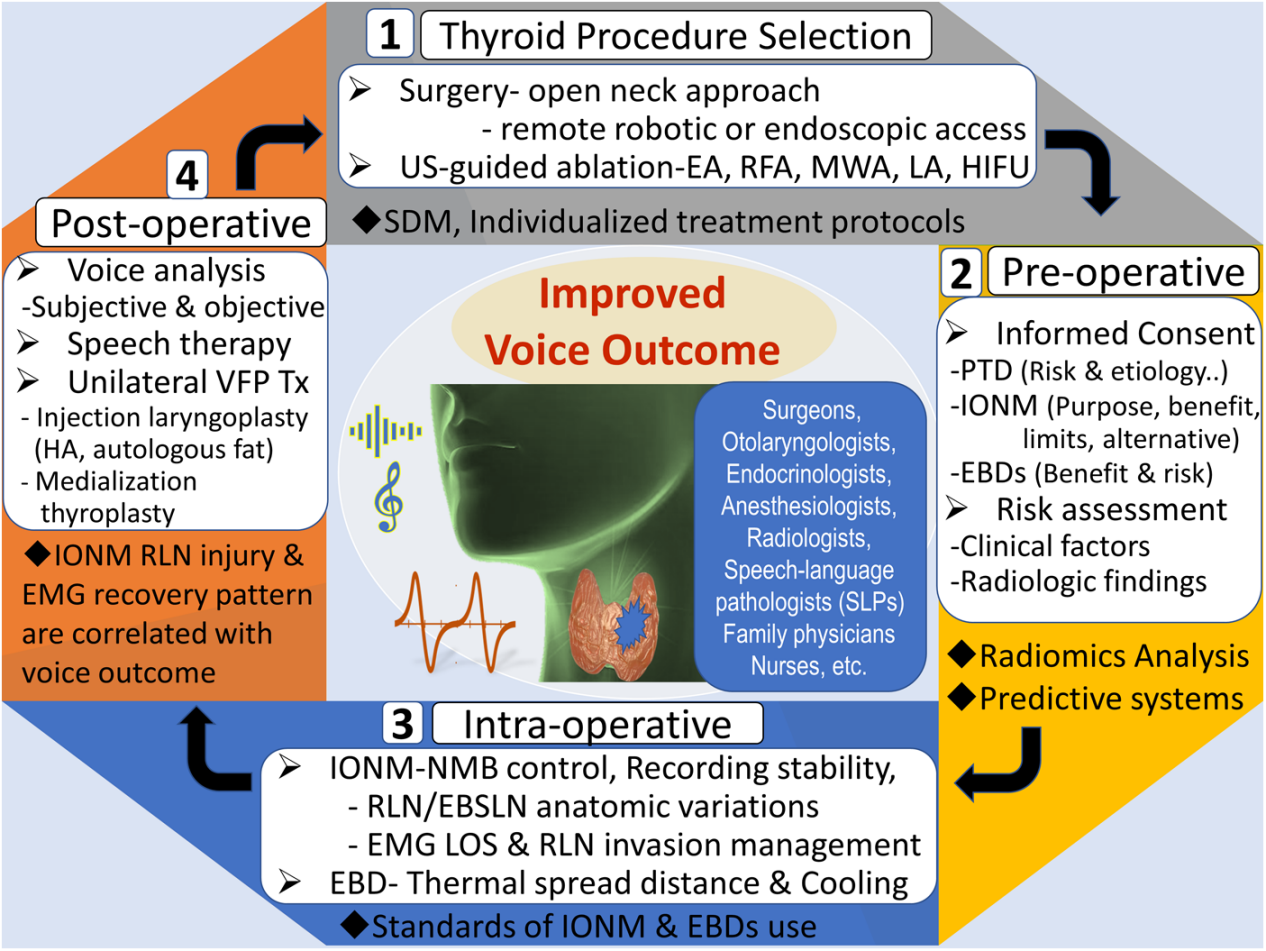
The use of a multifaceted approach to improve voice outcomes after thyroid surgery and ultrasound-guided ablation procedures. US, ultrasound; EA, ethanol ablation; RFA, radiofrequency ablation; MWA, microwave ablation, LA, laser ablation; HIFU, high-intensity focused ultrasound; SDM, shared-decision making, PTD, post-thyroid procedures dysphonia; IONM, intraoperative neural monitoring; EBDs, energy-based devices; NMB, neuromuscular block; RLN, recurrent laryngeal nerve; EBSLN, external branch of superior laryngeal nerve; EMG, electromyography; LOS, loss of signal; VFP, vocal fold paralysis; HA, hyaluronic acid.

Post-thyroid procedures dysphonia (PTD) is a common complaint of patients that not only affects the performance of professional voice users but also causes a decline in the quality of life of nonprofessional voice users (4). Possible causes of PTD include intra- procedure injury to the recurrent laryngeal nerve (RLN) or the external branch of the superior laryngeal nerve (EBSLN); vascular congestion; laryngeal edema; surgical trauma to the cricothyroid muscle or to the cricoarytenoid joint; endotracheal intubation-related trauma; surgical adhesions; strap muscle injury; and pain or psychological distress (5–8). Given the worldwide diffusion of thyroid procedures and the worldwide growing interest concerning the medico-legal implications of PTD and vocal fold paralysis (VFP), this special issue of Frontiers in surgery concerning the research topic “Improving Voice Outcomes after Thyroid Surgery and Ultrasound-guided Ablation Procedures” included several studies focusing on the patient's voice issues when undergoing thyroid surgery or US-guided ablation procedures during the preoperative, intraoperative, and postoperative period (Figure 1).

Papillary thyroid carcinoma (PTC) is the most common histological subtype of thyroid cancer, and surgery remains the mainstay of treatment. In PTC with thyroid capsule invasion (TCI), extrathyroid invasion (ETE), and central lymph node metastasis (CLNM), the risk of RLN injury is increased during surgery. Therefore, accurate preoperative evaluation TCI, ETE, or CLNM for PTC is important to determine surgical strategies and improve voice outcomes. Wu et al. reported combined models based on machine learning incorporated with CT radiomics features and the clinicoradiological risk factor and proved the model could be an effective, non-invasive, and safe tool for the preoperative prediction of TCI in PTC. In the study by Liu et al., the authors investigated the impact of Hashimoto's Thyroiditis (HT) on the predictive risk factors of CLNM in PTC and suggested that different predictive systems should be used for HT and non-HT patients to have a more accurate evaluation of central lymph node and determine the appropriate scope of lymph node dissection. In addition, Wang et al. proposed a risk assessment system for CLNM in papillary thyroid microcarcinoma (PTMC) of stage cN0 and explored its application value in clinical practice.

Compared to surgery for thyroid cancer, there has been a growing interest in developing the minimally invasive US-guided ablation treatment for benign thyroid nodules. Yan et al. constructed a nomogram to predict regrowth in patients with benign thyroid nodules after RFA with good discrimination and calibration capabilities. Patients with a high score had an increased probability of nodule regrowth and were potential candidates for additional ablation or surgical treatment. This reliable prognostic nomogram can guide the physician in stratifying patients and provide precise guidance for individualized treatment protocols and improve voice outcomes.

In the review article by Pace-Asciak et al., surgical and nonsurgical techniques for minimizing and treating PTD caused by an open approach or remote access thyroidectomy and RFA were introduced. Among the techniques for improving voice outcomes, intraoperative neural monitoring (IONM) has now gained widespread acceptance in the international community as an adjunct to visual nerve identification of the RLN and a useful tool for the external branch of superior laryngeal nerve (EBSLN) mapping during thyroid surgery (9). Wu et al. proposed the International Neural Monitoring Study Group (INMSG) consensus statement, which outlines general and specific considerations as well as recommended criteria for informed consent for the use of IONM. This consensus statement can assist surgeons and patients in the processes of informed consent and shared decision-making before thyroid surgery.

Teamwork between surgeons and anesthesiologists plays an important role in successful IONM. The utilization of neuromuscular blocking (NMB) agents facilitates tracheal intubation for general anesthesia. Once tracheal intubation is complete, the degree of NMB turns into a key factor for EMG signaling during IONM. Proper NMB management through the timing and dosage of reversal agents such as sugammadex and neostigmine are undergoing increasing amounts of investigations (10). Lu et al. proposed a useful clinical surgeon-centered sugammadex protocol according to NMB degree (0.5 mg/kg for deep block and 0.25 mg/kg for others) that provided high IONM quality and adequate surgical relaxation. Recently, Lu et al. also explored the feasibility of neostigmine (0.04 mg/kg) timely reversing NMB by both cisatracurium (0.2 mg/kg) and rocuronium (0.6 mg/kg) in a porcine model. These clinical and experimental studies can expand the options for precision NMB management during monitored thyroidectomy to improve vocal outcomes.

During IONM, the major limitation of the EMG tube recording system is the difficulty in maintaining stable contact between tube electrodes and vocal folds during surgical manipulation. Liu et al. reviewed the major recent developments of newly emerging transcartilage, percutaneous, and transcutaneous anterior laryngeal recording techniques used in IONM and highlighted their contribution to improved voice outcomes in modern thyroid surgery.

With the application of IONM, Aygun et al. analyzed the clinical and anatomical factors that affect RLN injury and reported that the RLN–inferior thyroid artery (ITA) relationship, extralaryngeal branches, and entrapment of the RLN at the Berry ligament are important factors affecting the development of postoperative VFP. This study concluded that revealing anatomical features with IONM and careful dissection can contribute to the risk reduction of PTD. Chiu et al. analyzed the intraoperative EMG recovery patterns and outcomes after RLN traction injury during IONM. The result shows different recovery patterns have different vocal cord function outcomes, and elucidating the recovery patterns can assist surgeons in intraoperative decision-making and postoperative management.

In addition to the IONM, another important technological advance in thyroid surgery in recent years is the introduction and development of energy-based devices (EBDs). The use of EBDs has many advantages such as reduced blood loss, lower rate of post-operative hypocalcemia, and shorter operation time. However, EBDs generate high temperatures that can cause iatrogenic thermal injury to the RLN by direct or indirect thermal spread. Wang et al. reviewed relevant medical literature and compares the safety parameters, such as safe activation distance and cooling time, between different types of EBDs used for thyroid surgery When using EBDs near the RLN in thyroid surgery, surgeons can adopt these safety parameters and follow the standard procedures to avoid laryngeal nerves or soft tissue injuries to improve the postoperative voice outcomes.

Unrecovered VFP and subjective voice impairment after thyroid surgery causes extreme distress in patients. Huang et al. investigated the correlations between IONM findings and voice outcomes in patients with impaired VFM after thyroid surgery. The result showed that severe type RLN injury (e.g., thermal injury or injury causing EMG decrease >90%) raises the risk of unrecovered VFM and moderate/severe long-term postoperative subjective voice impairment. This study suggested that objective voice parameters (e.g., pitch range) can be used as prognostic indicators not only to enable surgeons to earlier identify patients with low voice satisfaction after surgery, but also to enable the implementation of interventions sufficiently early to maintain quality of life.

High-pitched voice impairment (HPVI) is not uncommon in patients without RLN or EBSLN injury after thyroidectomy, which is commonly caused by fibrosis and limited movement of the strap, lateral extralaryngeal, or cricothyroid muscles. Huang et al. evaluated the correlation between subjective and objective HPVI in patients after thyroid surgery. The result showed factors that affect a patient's subjective HPVI are complex, and voice stability (Jitter and Shimmer) is no less important than the Fmax level. Therefore, the authors suggested that maximum frequency (Fmax) and Index of voice and swallowing handicap of thyroidectomy (IVST) scores should be interpreted comprehensively, and surgeons and speech-language pathologists (SLPs) should work together to identify patients with HPVI early and arrange speech therapy for them.

Concerning the treatment in patients with unilateral VFP after thyroidectomy, Wen et al. reported a retrospective case series in a tertiary teaching hospital to compare the clinical outcomes between different treatment options, including voice therapy (VT), hyaluronic acid (HA) injection, autologous fat injection (FI), and medialization thyroplasty (MT). The results revealed that VT, HA, FI, and MT can all improve the voice outcomes of patients and suggested that the optimal treatment approach should be individualized according to the patient's preference, vocal demand, and the interval between thyroidectomy and intervention. Finally, Liao et al., performed a literature review using the PubMed, Medline, and EMBASE databases to determine the timing, materials, methods, and outcomes of injection laryngoplasty (IL) for unilateral VFP after thyroid surgery. This state of art review supported that IL could improve the voice outcome for unilateral VFP after thyroid surgery, and the autologous fat remains a good augmentation material with a potential longer lasting effect.

In conclusion, this special issue comprises highly qualified papers, which provide novel and comprehensive information for clinicians involved in managing thyroid tumor patients, which includes surgeons, otolaryngologists, endocrinologists, radiologists, internists, speech-language pathologists, family physicians, and other primary care providers, anesthesiologists, nurses, and others. The increasing interest in prevention rather than treatment of PTD may lead to a greater increase in the cost-effectiveness of thyroid tumor treatment, a reduction in procedure-related morbidity, and better health-related quality-of-life (Figure 1).
